# Effects of perceptual-cognitive tasks on inter-joint coordination of soccer players and ordinary college students

**DOI:** 10.3389/fpsyg.2022.892118

**Published:** 2022-10-05

**Authors:** Yuanyuan Ren, Cenyi Wang, Aming Lu

**Affiliations:** School of Physical Education and Sport Science, Soochow University, Suzhou, China

**Keywords:** cognition, inter-joint coordination, performance, dual-tasks, landing

## Abstract

Perceptual-cognitive tasks play a pivotal role in performing voluntary movements, which is crucial for good performances among soccer players. This study explored the effect of perceptual-cognitive tasks on the inter-joint coordination of soccer players and college students during landing. The classic multiple objective tracking (MOT) task was used to simulate the perceptual-cognitive task under a sports environment. Fifteen soccer players (age: 20.1 ± 1.5 year, height: 181.4 ± 7.4 cm, weight: 75.4 ± 10.7 kg) and twenty ordinary college students (age: 20.0 ± 2.3 years, height: 177.9 ± 4.9 cm, weight: 71.6 ± 9.9 kg) were enrolled to the study. Participants in the two groups were subjected to a single task (landing task) and dual-task (MOT task and landing task). Coordination and variability indicators were recorded using a Vicon infrared motion capture system and a force measuring platform. The results showed that the mean absolute relative phase of hip and knee joint (MARP_hip-knee_), deviation phase of hip and knee joint (DP_hip-knee_), and deviation phase of knee and ankle joint (DP_knee-ankle_) of the two groups under the dual-task were significantly different compared with the parameters when participants were subjected to the single task. The dual-task had higher effect size on DP_hip-Knee_ and MARP_hip-knee_, indicating that dual-task had a greater impact on coordination of the hip and knee joints. DP_hip-knee_ and DP_knee-ankle_ of ordinary students were more extensive relative to those of the soccer players, and hip joint stiffness (*K*_hip_) for ordinary students was lower than that of the soccer players under the different tasks. These findings implied that the perceptual-cognitive task markedly affected the inter-joint coordination of soccer players and college students, mainly by impairing the hip and knee coordination. Although there is less variability in lower extremity coordination patterns of soccer players compared to college students, the MOT task still affects their coordination ability.

## Introduction

Perceptual-cognitive abilities in interactive sports are correlated with the visual attention features and the dynamic tracking tasks conducted in sports. Soccer players are required to focus on the position of the ball, teammate and the opponent concurrently high perceptual-cognitive abilities are important. The ability to divide attention and dynamically track multiple moving objects is known as Multiple Object Tracking (MOT) task. It plays a crucial role in performing voluntary movements, and highly contributes to success in team sports. MOT tasks can be used for effective evaluation and simulation of perceptual-cognitive tasks in dynamic environments of athletics (Ehmann et al., 2021).

Several laboratory studies have reproduced such dynamic situations using the MOT task paradigm, which is the main approach used for studying interactions between cognitive processing and motor behavior. The biomechanical effects of 3D-MOT on lower extremity landings have been extensively explored in recent studies, and the findings indicated that combination of perceptive-cognitive task with muscle fatigue alters knee kinematics and increases the risk of anterior cruciate ligament rupture (Mejane et al., 2019). MOT tasks can reduce the postural stability of soccer players, as well as increase the risk of injury of soccer players (Ren et al., 2021). Findings from a previous research showed that soccer players had a 14.4% higher injury rate during matches compared with the rate of injury observed during regular training (Romeas et al., 2016), which was partially attributed to MOT tasks undertaken during matches. Differences in attention allocation and perceptual-cognitive ability between soccer players and ordinary students have been observed in the past, and various results indicate that soccer players are proficient in cognitive tasks (Alves et al., 2013; Qiu et al., 2018). A study was conducted to compare the cognitive abilities of elite athletes, ordinary athletes, and healthy adults. The results showed that elite athletes had better performance in two control tasks and a visuospatial attention task compared with ordinary athletes and healthy adults (Alves et al., 2013). In addition, previous findings indicated that high-performance athletes who had been trained for a long time had better performance after subjection to tracking multiple targets with different attention loads relative to the performance of non-athletes (Qiu et al., 2018). The effect of perceptual-cognitive training on improving cognitive function and specific motor performance in humans has been explored in recent studies. Long-term MOT training improves athletic performance and enhances the winning ability during soccer matches (Romeas et al., 2016). Participants in a video-based perception training group in a study conducted previously showed improved decision-making, fewer recall errors, improved shooting, and other specialized skills after 4 weeks of intervention compared with that of the non-intervention control group (Gabbett et al., 2008). This indicates that resource allocation processes involved in complex motor environments and visual attention overlap in brain regions, and long-term training causes plasticity changes in brain activities (Dahlin et al., 2008).

Soccer is a popular and complex sport that requires perceptual-cognitive activities such as visual attention (Alvarez and Cavanagh, 2005). High requirements for coordination between limbs, good limb coordination, and stability are highly associated with excellent performance of soccer players (Zhang et al., 2021). Inter-joint coordination mode of the lower extremity is a major indicator for evaluating lower extremity load and sports injury during dynamic movement (Schoner and Kelso, 1988; Kim et al., 2019; Sinsurin et al., 2020; Zhang et al., 2021). Joint coordination during landing is mainly reflected through joint stiffness, and joint stiffness can be used to evaluate soft tissue injury in the lower extremity (Li et al., 2021). These findings imply that inter-joint significantly affects the performance of players. Coordinated activities can reduce the load on joints during movement by improving dynamic stability of the participant, resulting in efficient and accurate functional movement. The landing movement, which is a common movement type in sports, is a frequent movement in soccer activities such as heading of a soccer ball. Approximately 60% of injuries are associated with landing movements (Rahnama et al., 2002), thus landing movement is used to explore effective control strategies for preventing injuries. Studies conducted in the past reported possible interaction between perceptual-cognitive tasks and motor control, however, the effects of perceptual-cognitive tasks on inter-joint coordination and injury risk have not been fully elucidated. In addition, studies have not fully explored whether dual tasks (especially MOT tasks) affect the entire lower extremity, and possible differences in motor control and regulation modes in soccer players and college students should be elucidated.

Therefore, the purpose of this study was to evaluate the effects of MOT tasks interference on inter-joint coordination during landing. In addition, the motor control of soccer players and ordinary college students under different tasks was explored by comparing the effects of MOT tasks on the lower limb coordination patterns of the participants. The hypothesis formulated for the study was that attention processes involved in MOT tasks are important factors resulting in an increase in individual impairment and causes changes in lower extremity coordination mode. Furthermore, it was hypothesized that well-trained soccer players would exhibit better inter-joint coordination under interference of different tasks compared with ordinary college students who had not undergone long-term training. This study provides an important reference for further studies to design strategies for preventing injuries in individuals involved in sports.

## Materials and methods

### Participants

A total of 35 male participants from Soochow University were recruited to the study through posters. The participants included 15 well-trained players from a high-level soccer team in the university and 20 college students who had no experience participating in major athletic competitions or training. The inclusion criteria for the study were as follows: (1) Participants who had no history of diseases or injuries affecting the lower extremity joints within approximately half a year and were able to complete the experiment; (2) Subjects who had not undertaken strenuous exercise within 24 h before the test. Personal basic information was obtained using a questionnaire and participants who did not meet the inclusion criteria were excluded from the study (Table 1).

**Table 1:** Basic information of the study participants (mean ± SD).

Information	Players	Students	*t*-value	Value of *p*
Age (yr)	20.1 ± 1.5	20.0 ± 2.3	0.09	0.93
Height (cm)	181.4 ± 7.4	177.9 ± 4.9	1.57	0.13
Weight (kg)	75.4 ± 10.7	71.6 ± 9.9	0.98	0.34

yr, year; cm, centimeter; kg, kilogram.

### Materials and study equipment

**Figure 1:**
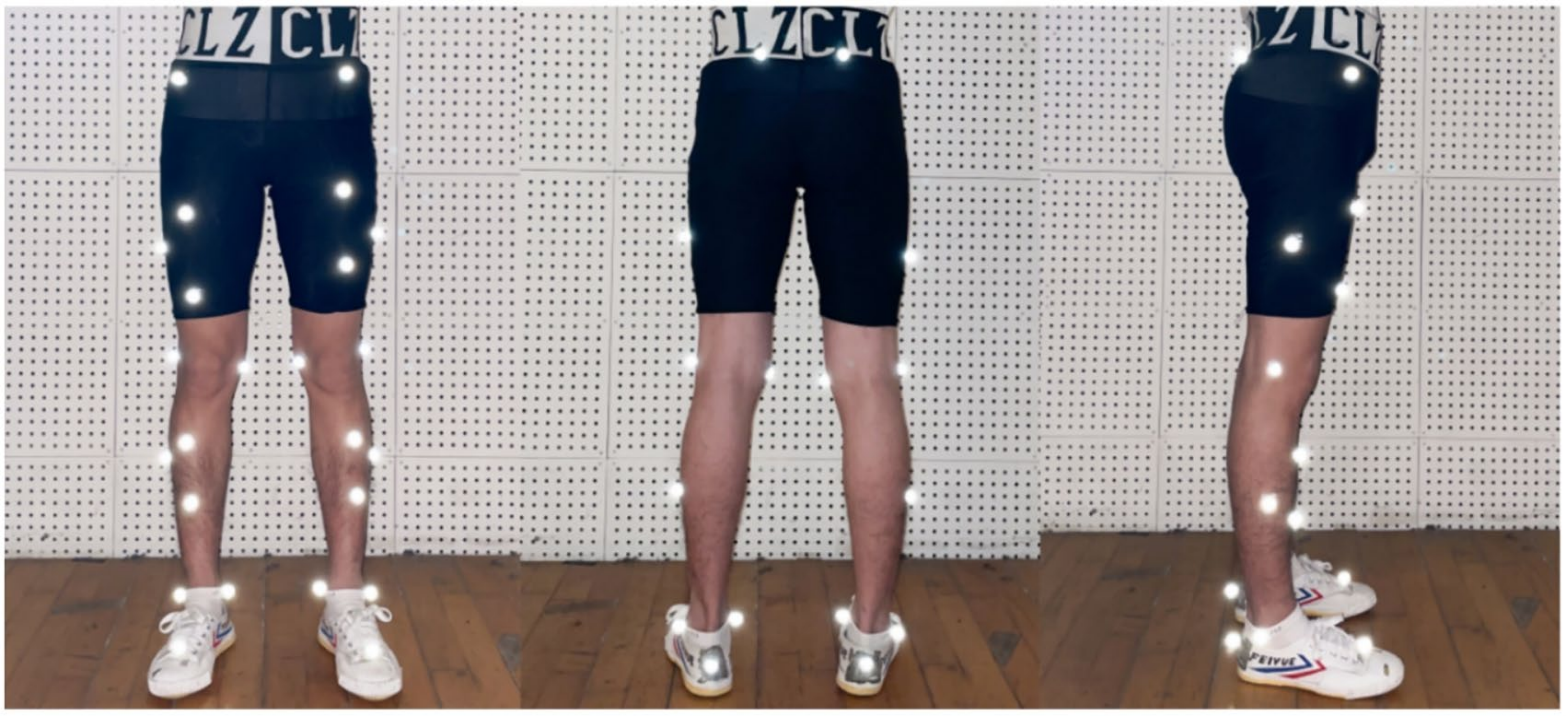
Marker sticking positions.

A 3D projector and a screen were placed directly in front of the participants who were requested to perform perceptual-cognitive tasks. MOT tasks were presented as self-written programs, which were projected to a 220 cm screen using the projector. The distance between the participant and the screen was 130 cm (for MOT and landing tasks). Landing tests were performed on a force measuring platform using a large pressure sensor (Kistler company, Switzerland, Model 9287B) at a sampling rate of 1,000 Hz, to allow online detection of ground reaction force and determination of balance characteristics. Vicon infrared motion capture system with 8 infrared cameras (Vicon, Inc., Oxford, United Kingdom) with a sampling rate of 100 HZ and a Vicon plug-in gait model were used to obtain kinematic data on the sagittal plane. The model markers were placed at the following positions: bilateral anterior superior iliac spines, bilateral posterior superior iliac spines, bilateral proximal thigh (1/3 of the distance from the hip to the knee), bilateral distal thigh (2/3 of the distance from the hip to the knee), mid-lateral thigh (at the middle of the lateral thigh from the hip to the knee), lateral condyles of both knees, bilateral proximal calf (1/3 of the distance from the knee to the ankle), bilateral distal calf (2/3 of the distance from the knee to the ankle), the lateral middle of the calf (at the middle of the lateral calf from the knee to the ankle), bilateral malleolus, bilateral calcaneus, and bilateral second phalanges (Figure 1). All the pasting work of model markers was only conducted by a trained professional to reduce experimental error. Data were collected from 2019 to 2021.

### Study design

**Figure 2:**
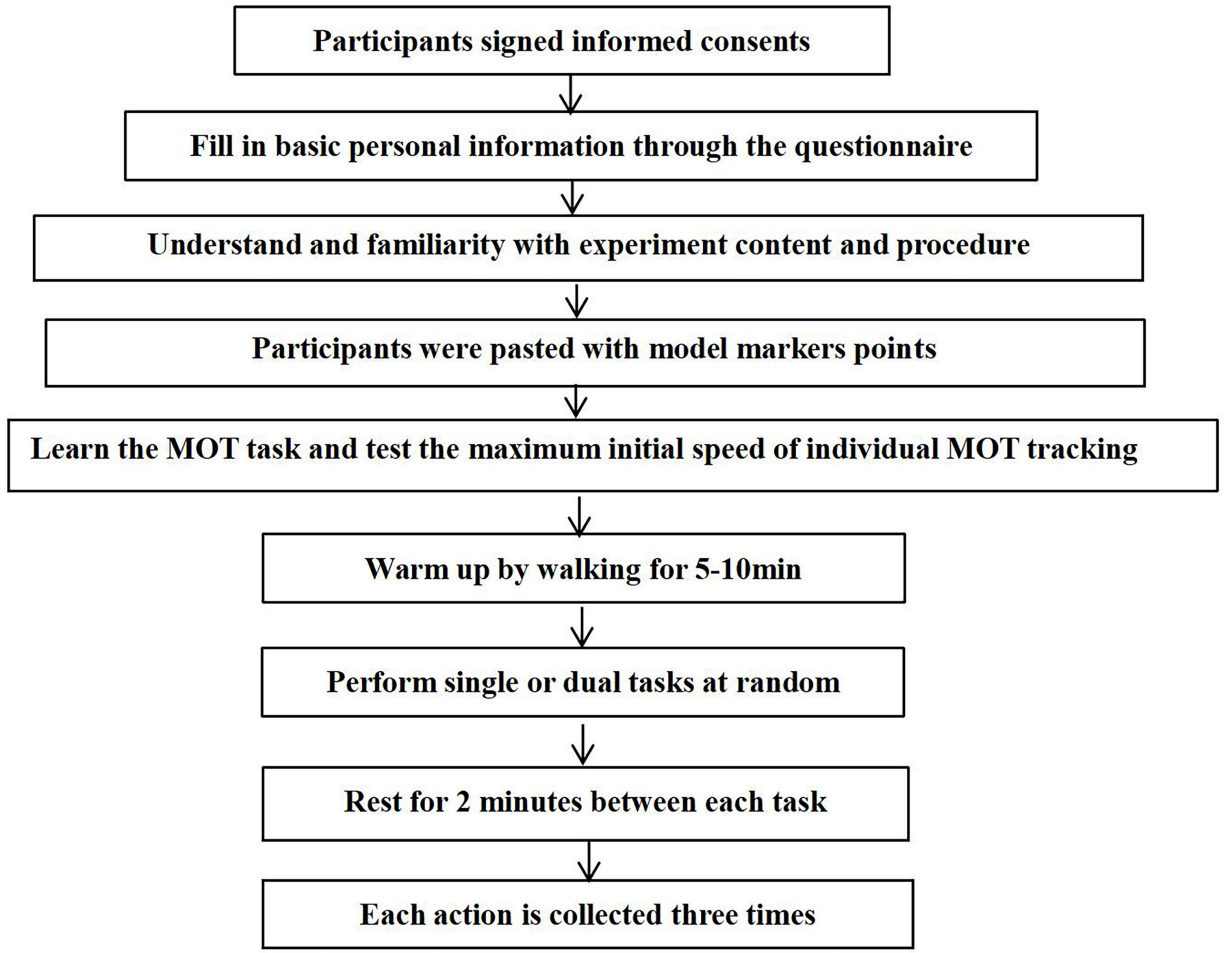
Schematic representation of the study design.

All participants signed an informed consent form and provided details on personal information by filling a questionnaire. Subsequently, participants were briefed on the experiment process and action, and positions were labeled on their lower limbs. The initial maximum tracking speed of MOT tasks was then determined for each participant and the standardized speed was recorded. Further, participants warmed up by walking at 4 km/h for 5–10 min on a treadmill. Participants were then randomly assigned to single task (ST) or dual-task (DT) trials. The experimental protocol comprised ST and DT dimensions. Participants completed the landing movement in ST and completed synchronously the MOT task and landing movement in DT. Each participant followed the experiment procedure (Figure 2).

### Conducting of MOT and landing tasks

**Figure 3:**
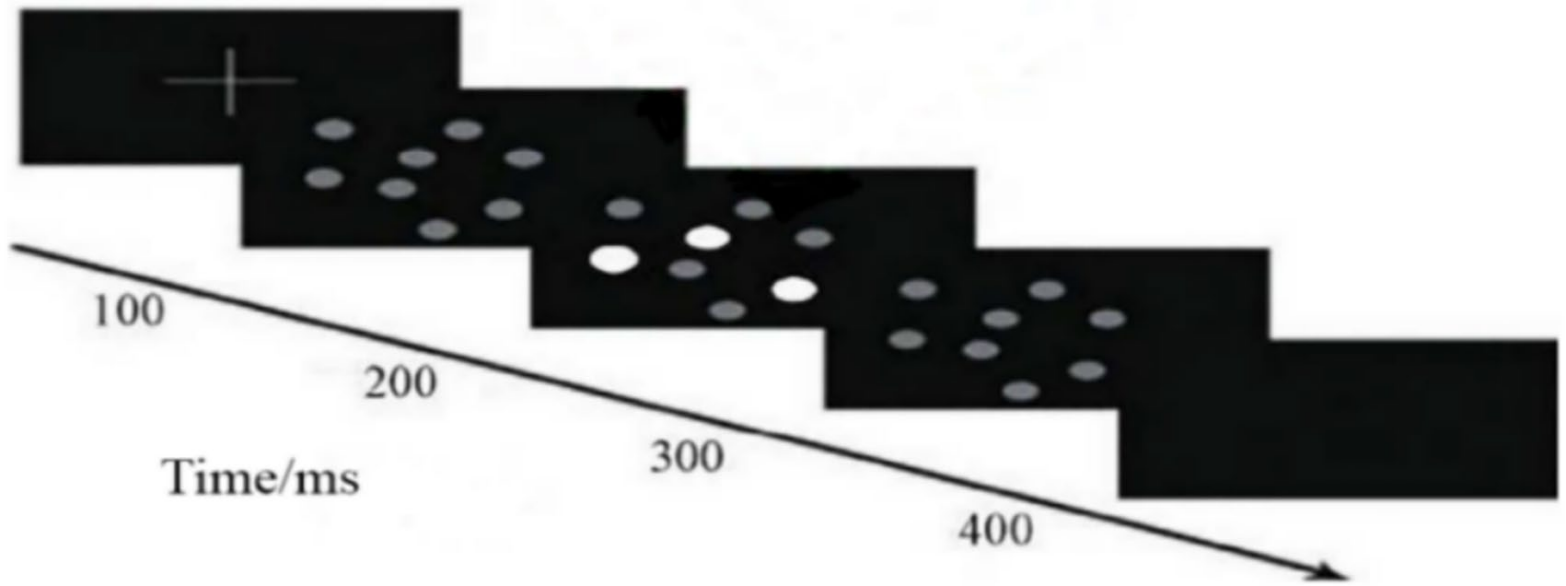
A representation of the multiple objective tracking (MOT) task experimental process.

Eight identical spheres were adopted in conducting MOT tasks (Figure 3). These spheres interacted dynamically in a three-dimensional space (bounced off each other, broke away from the virtual three-dimensional volume boundary, and blocked each other). The task was divided into four steps as follows: (a) Eight randomly organized identical spheres were presented on the screen; (b) The color of three target spheres changed; (c): The color of all the spheres changed to the original color and the spheres moved randomly around the screen at the same speed; (d): The participants were requested to point out the position of the three target spheres on the screen independently at the end of the task. The moving speed of target spheres were standardized according to the participant’s threshold to ensure that all participants received the same MOT task load (Mejane et al., 2019). The standardization process was conducted as follows: The task was first presented at a random speed and the speed of the next MOT task was increased when the participant correctly identified three target spheres. The speed of the subsequent MOT task was decreased if the three target spheres were not correctly identified. The speed of spheres was gradually adjusted until the participant’s initial maximum tracking speed was achieved. A standardized speed corresponding to 30% of the initial maximum tracking speed was chosen to ensure that all participants were able to perform dual tasks (Ren et al., 2021) and to minimize differences in tracking speed among participants. Movement of the MOT task on the screen was used as a cue for landing movement and was applied to ensure that both tasks were completed simultaneously. A failure was recorded when participants focused only on one of the two tasks and finished the tasks sequentially thus the test was repeated. Participants signed an informed consent form prior to taking part in the experiment, and each participant was requested to familiarize themselves with the MOT task and fully understand it (a correct rate of 90% or more) in advance to minimize learning effects.

In this study, each participant was requested to stand on a 40 cm platform with his hands on his hips and feet positioned upright. The participant used his dominant leg to step forward after receiving the “start” signal; subsequently, the participant leaned forward and fell vertically from the step without initial speed with the “toe-heel” landing mode. Each action was conducted in triplicates and the average value was calculated.

### Data collection and analysis

The indicators of joint stiffness and coordination of the dominant lower extremity of the participants were analyzed in this study. Inter-joint coordination during landing mainly reflects joint stiffness, and reduction of joint stiffness is a potential risk factor for soft tissue injury (Li et al., 2021). Coordination-related parameters in this study included continuous relative phase (CRP), mean absolute relative phase (MARP), and deviation phase (DP), which were recorded using Vicon infrared motion capture system during ST and DT. Inter-joint coordination was evaluated to determine essential timing and sequencing of the neuromuscular system control over biomechanical degrees of freedom. Variability of coordination indicated adaptability of the neuromuscular system control. The landing process was defined as the period from touchdown (the moment vertical ground reaction ≤10 N was achieved) to the time maximum knee flexion was attained (Zhang et al., 2021). The duration of the landing process was normalized to 0–100%, with 0% representing the beginning of the landing process and 100% representing the end of the landing process. An inhouse MATLAB script (MatlabR2018b, MathWorks, Natick, MA, United States) was used for calculation of the indicators in this study.

CRP was used to describe the inter-joint coordination’s mode and variability. MARP and DP were used to describe coordination (in-phase or anti-phase coupling) and variability between joints. The angle (*θ*) and angular velocity (*ω*) were normalized to minimize the effect of different movements in amplitudes and frequencies using formula (1) presented below. In the current study, the maximum angle of the sagittal plane of the lower extremity joints was normalized as “+2,” the minimum angle was normalized as “0,” and the angular velocity (*ω*) was normalized to the maximum value (Sheikhi et al., 2021).


(1) $$\omega_{\left(i\right)\mathrm{norm}}=\omega /\max\left[\max\left(\omega_{i}\right),\max\left(-\omega_{i}\right)\right]$$

(2) $$\theta_{\left(i\right)\mathrm{norm}}=2*\left[\theta_{j}-\min\left(\theta_{i}\right)\right]/\max\left(\theta_{i}\right)-\min\left(\theta_{i}\right)$$

**Figure 4:**
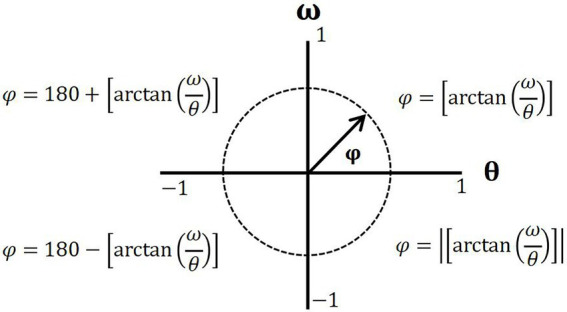
Standardization of the four-quadrant phase diagram.

The relative phase angle ( φ ) of the different joints was calculated using formula (2) and standardized according to the phase diagram of the four quadrants (Figure 4). CRP values range from −180° to 180°, where a CRP close to 0° indicates that the two joints move “in-phase,” whereas a CRP close to 180° or −180° represents an “anti-phase” motion of the two joints (Sheikhi et al., 2021).

CRP values were calculated based on the difference between the distal joint and the proximal joint using formula (3) presented below. A positive value of CRP indicated that the proximal joint dominated the distal joint, whereas a negative value of CRP indicated that the distal joint dominated the proximal joint. MARP represents the mean value of each joint’s phase angles on the curve and is calculated using formula (4), and DP indicates the standard deviation of each point on the overall curve and is calculated using formula (5).


(3) $$CRP=\varphi_{\mathrm{distal}}-\varphi_{\mathrm{proximal}}$$

(4) $$\mathrm{MARP}=\frac{\sum_{i=1}^{n}|\varphi |{}_{i}}{n}$$

(5) $$DP=\frac{\sum_{i=1}^{n}\;SD_{i}}{n}$$

The joint stiffness (*K*_joint_) was then calculated based on the net torque change in the sagittal plane (Δ*M*_joint_) and displacement change of joint angle in the sagittal plane (Δ*θ*_joint_) during landing (Farley et al., 1998). The formula for calculation of joint stiffness is presented below:


(6) $$K_{\mathrm{joint}}=\Delta M_{\mathrm{joint}}/\Delta \theta_{\mathrm{joint}}$$

### Statistical analysis

SPSS statistics software (22.0, IBM Inc., Chicago, IL, United States) was used for statistical analysis. Shapiro–Wilk test was used to determine the normality of the data. Levene test was conducted to evaluate homogeneity of variance, and the studentized residuals method was used to determine presence of outliers. Mean (*M*) and standard deviation (SD) of the parameters were calculated and mixed repeated measure ANOVA was used to determine differences among variables with the group as the between-group factor (amateur soccer players and ordinary college students) and task as the within-group factor. Significance was set at *p* < 0.05.

## Results

### Changes in speed of spheres

The results showed that the average speed of the spheres was significantly different between the two groups of participants (Table 2). The initial maximum movement speed and standardized movement speed of the soccer players were higher than that of ordinary students.

**Table 2:** Changes in speed of spheres in different groups (mean ± SD).

Movement speed (°/s)	Players	Students	*t*-value	Value of *p*
Initial maximum speed	14.60 ± 2.44	12.77 ± 2.83	−2.092	0.043
Standardized speed	4.42 ± 0.81	3.83 ± 0.85	−2.179	0.035

*K*_hip_, the hip joint stiffness; *K*_knee_, the knee joint stiffness; *K*_ankle_, the ankle joint stiffness; ST, single task; DT, dual task.

### Changes in inter-joint coordination

**Figure 5:**
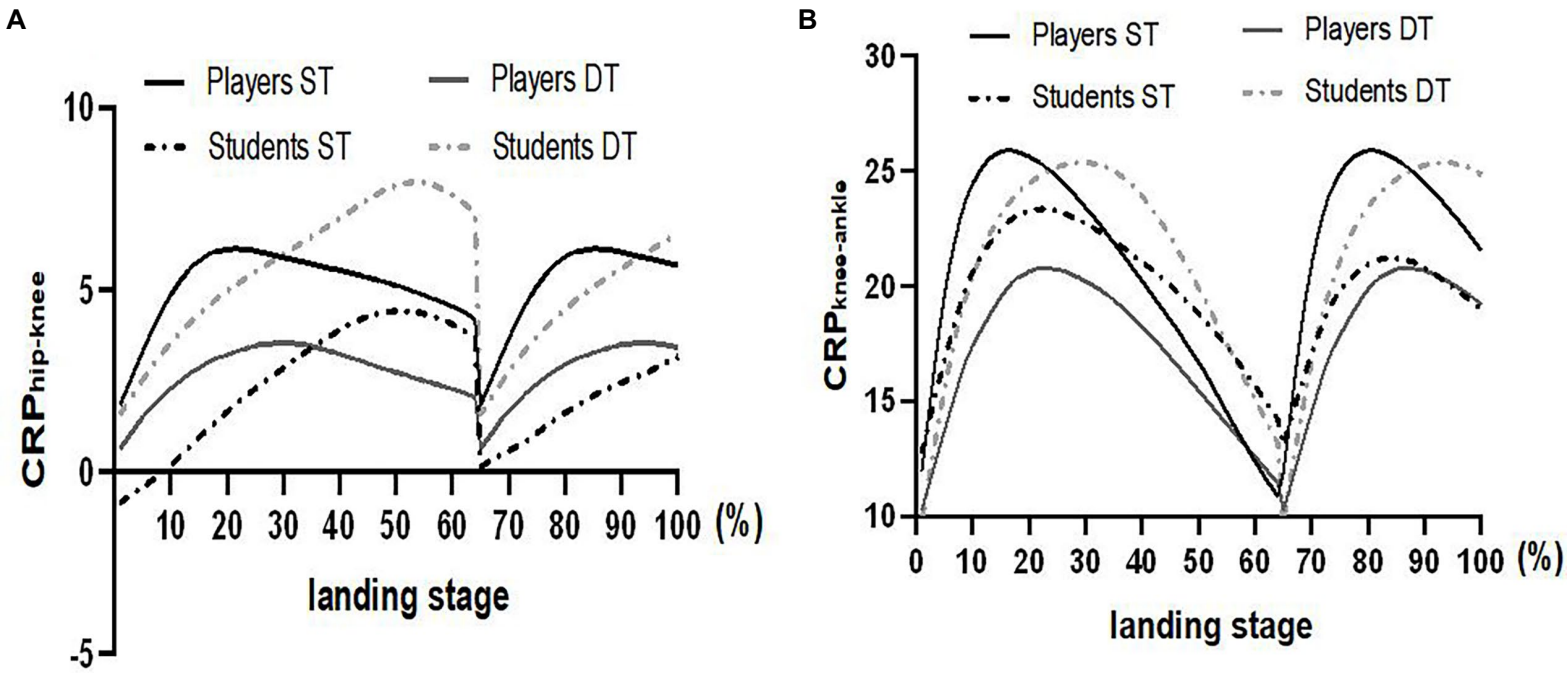
Changes in continuous relative phase (CRP) during landing.

The CRP change curves of hip, knee, and ankle joints of soccer players and ordinary college students under two task conditions are presented in Figure 5. The CRP_hip-knee_ and CRP_knee-ankle_ values of the soccer players group in DT were significantly reduced than in ST; CRP_hip-knee_ values of the ordinary student group in DT were significantly higher than in ST.

**Figure 6:**
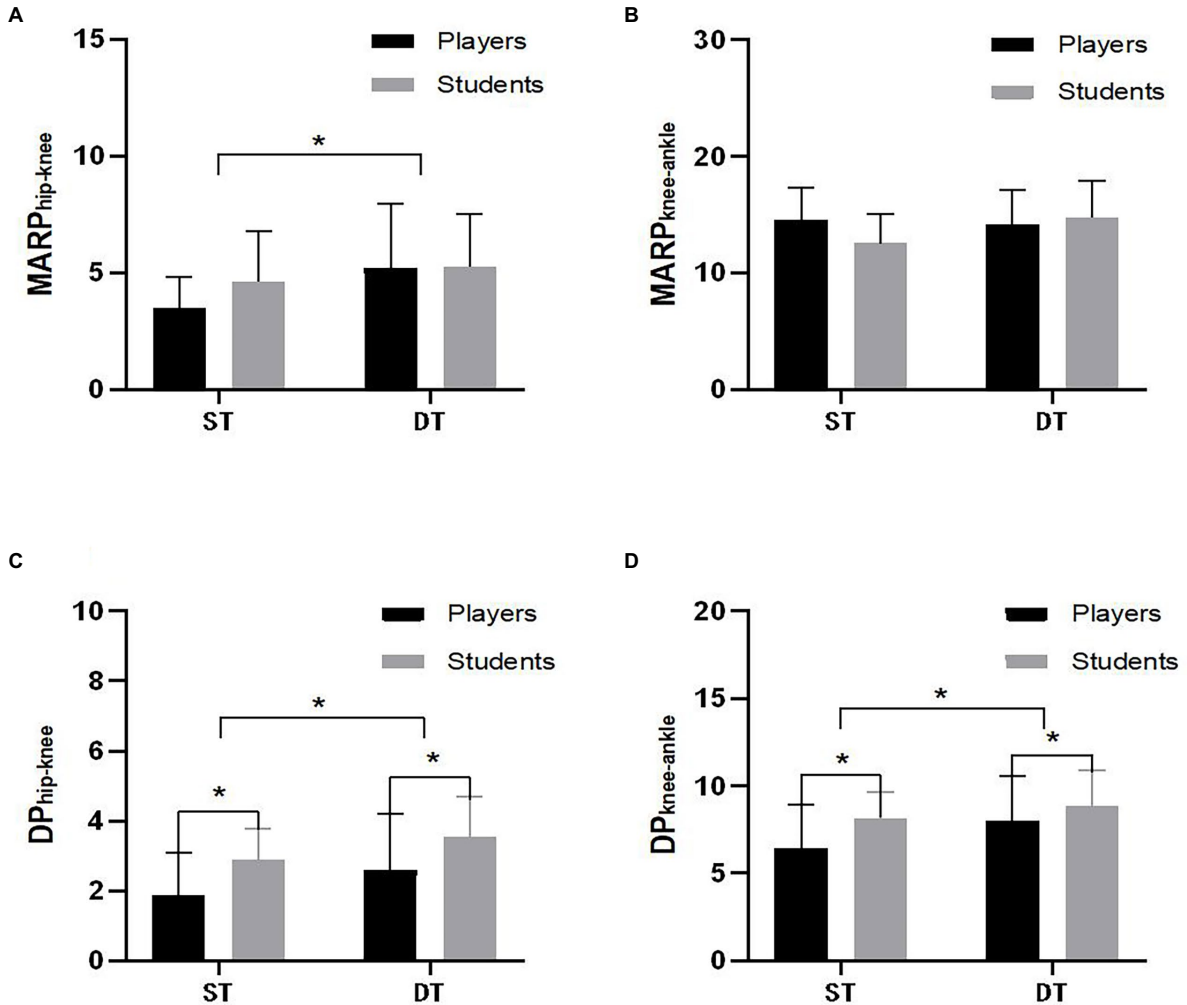
Changes in mean absolute relative phase (MARP) and deviation phase (DP) in the two groups under the two tasks.

The MARP and DP changes under the two task conditions are presented in Figure 6. The MARP_hip-knee_, DP_hip-knee_ and DP_knee-ankle_ changes in DT were significantly different relative to the values of the changes in ST (*F*_1,37_ = 4.362, *p* = 0.041, partial *η*^2^ = 0.070, Cohen’s *d* = −0.523; *F*_1,37_ = 4.831, *p* = 0.032, partial *η*^2^ = 0.077, Cohen’s *d* = −0.526; *F*_1,37_ = 4.276, *p* = 0.043, partial *η*^2^ = 0.069, Cohen’s *d* = −0.498). In addition, dual-task had a greater effect size on DP_hip-Knee_ and MARP_hip-knee_, but a smaller effect size on DP_knee-ankle_, indicating that dual-task had a higher effect on coordination of the hip and knee joints than single-task for both soccer players and college students. Moreover, the DP_Hip-Knee_ and DP_Knee-ankle_ of ordinary college students during landing were significantly larger relative to that of soccer players (*F*_1,27_ = 9.888, *p* = 0.003, partial *η*^2^ = 0.146, Cohen’s *d* = 0.798; *F*_1,27_ = 5.279, *p* < 0.025, partial *η*^2^ = 0.083, Cohen’s *d* = 0.586). It showed that the DP_Hip-Knee_ and DP_Knee-ankle_ values of college students were significantly higher than those of the soccer players in ST or DT.

### Changes in joint stiffness

The *K*_hip_ of soccer players was significantly larger in DT than in ST, the *K*_hip_ of college students was also significantly larger in DT than in ST during landing (*F*_1,27_ = 4.226, *p* = 0.044, partial *η*^2^ = 0.068, Cohen’s *d* = −0.549), and the effect size of *K*_hip_ was moderate. It shows that MOT tasks cause changes in lower extremity mode such as joint stiffness (Table 3). Moreover, No statistically significant difference was observed in *K*_hip_ of participants under the two tasks (*p* > 0.05).

**Table 3:** Changes in joint stiffness in the two groups under the two tasks (mean ± SD).

	Players		Students	
Joint stiffness	ST	DT	ST	DT
*K*_hip_	0.222 ± 0.060	0.279 ± 0.060	0.141 ± 0.114	0.168 ± 0.106
*K*_knee_	0.015 ± 0.010	0.014 ± 0.010	0.014 ± 0.025	0.011 ± 0.098
*K*_ankle_	0.041 ± 0.020	0.045 ± 0.020	0.117 ± 0.217	0.108 ± 0.208

*K*_hip_, the hip joint stiffness; *K*_knee_, the knee joint stiffness; *K*_ankle_, the ankle joint stiffness; ST, single task; DT, dual task.

## Discussion

The purpose of this study was to explore whether MOT task interference affects inter-joint coordination during the landing task. The results showed that MARP_hip-knee_, DP_hip-knee_, and DP_knee-ankle_ value under DT were higher compared with the values under ST, indicating that MOT tasks negatively affected inter-joint coordination of soccer players and ordinary college students. This finding is similar to findings from previous research whereby the effects of attentional control on gait and inter-joint coordination were evaluated using a dual-task paradigm. The previous findings showed that attentional control modulated the ability to maintain gait control and regulated inter-joint coordination patterns when adapting to dual-task gait perturbations (Wang et al., 2021). The kinematic chain theory indicates that increase in MARP_hip-knee_ and DP_hip-knee_ during landing causes changes in the coordination pattern and variability of the lower extremity hip-knee joint. Studies report that coordination of the hip-knee joint determines the dynamic stability of the lower extremity (Sinsurin et al., 2020), and hip-knee joint coordination is a dominant kinematic variable for reducing the risk of injury (Leonard et al., 2021). Therefore, the increase of MARP_hip-knee_ observed in our study indicated that the “inverse” movement of the hip joint and knee joint increased over time. This increase indicated a significant contribution of the hip (proximal joint) to the movement of the participant under DT condition. Furthermore, the findings of the current study showed that dual tasks had a greater impact on hip and knee coordination, which agree with past findings that the hip joint is the primary stabilizer of lower extremity movement (Davis et al., 2019). Highly intense hip motion results in high motor instability. Therefore, the coordinated response of the hip—knee was explored in this study because it is a paramount kinematic variable for reducing the risk of injury. In addition, increase in DP_knee-ankle_ indicated an increase in variability of the knee-ankle joint (calf movement). Active calf movement can decrease the dynamic stability of the hip during coupling movement and change the load on the lower extremity joints during dynamic movement, resulting in increased occurrence of injuries. Other studies reported that distraction affects performance of female athletes when they passed the ball and completed a side-cut action simultaneously (Almonroeder et al., 2019), similar to the findings from our study. The results of the our study are inconsistent with previous findings (Qu and Hu, 2014), which indicated that younger adults choose whether to focus on cognitive tasks to adjust their motor coordination patterns based on the level of difficulty of the task. This difference can be attributed to differences in the types of tasks and loads used in the various studies. The sequential subtraction tasks used previously did not involve sensory perception. In addition, 3D-MOT tasks may not have the similar effects on individuals as other cognitive tasks. Therefore, further research should be conducted to explore the effects of different types of cognitive tasks on individuals.

A previous study reported that soccer players have to extract and process information after subjection to cognitive and sensory tasks especially when they take part in team sports (Romeas et al., 2016), which is consistent with the present findings. These specific cognitive functions (MOT tasks) decreased when participants were exposed to higher stress levels (during games or competitions), leading to a decrease in attention control ability (Romeas et al., 2016). A previous review article summarized various studies that reported the effects of distraction on landing activities with the findings indicating that distraction can impair performance in sports similar to the findings of the present study (Hughes and Dai, 2021). In addition, another past study indicates that complex cognitive task have a significant effect on jump-landing movement quality, which can be attributed to specific cognitive functions such as attention allocation (Schnittjer et al., 2021). Neurocognitive mechanism comprises a group of regions mainly located in the frontal and parietal regions of the cerebral cortex (precentral gyrus and paracentral gyrus). From the perspective of neurocognitive mechanism, the central motor command consists of a group of regions located primarily in the frontal and parietal regions of the cerebral cortex (precentral gyrus and paracentral gyrus), responsible for the control of muscle tissue by Beta cells from the superficial regions of the cerebral cortex into the corticospinal tract. These motor fibers cross first as they travel from the brain to the medulla and spinal cord before reaching the muscles. There is compelling evidence that activity in these motor areas may depend on attentional demands imposed by physical tasks (Bigliassi et al., 2016).

However, the MOT task, which is a high-level perceptual-cognitive interference, involves several cognitive processes, such as visual attention allocation (Trick et al., 2012). The effects observed can be attributed to the limited allocation of visual attention because the same neural network is implicated in controlling motor and cognitive functions. Notably, the MOT task requires allocation of a higher number of cortical resources, thus the dual task could have been affected resulting in changes in the motor system beyond conscious control. Results using theoretical capacity-sharing model explains this phenomenon (Pashler, 1994). In the present study, visual information played a key role in completion of the dual-task by the participants. Participants may have prioritized one task (MOT) by reducing self-control task (landing) because perceptual-cognitive tasks (MOT task) and motor-related demands (landing task) competed for frontal lobe resources. This affected postural control of the participants and impaired inter-joint coordination. Therefore, these results indicate that individual neuromuscular activity under the MOT task did not effectively control the challenging dual-task.

The results showed that the DP_Hip-Knee_ and DP_Knee-ankle_ values of college students were significantly higher than those of the soccer players in ST or DT, indicating that college students exhibited more inter-joint coordination variability compared with soccer players, while MOT tasks did not affect this finding. Previous studies have shown that excessive variation of lower extremity coordination mode could exacerbate impairment of neuromuscular control, resulting in abnormal biomechanical patterns and higher joint loads when subjects participate in sports (DiCesare et al., 2019). These results may reflect that soccer players exhibit less inter-joint coordination variability and have better coordination, and they perform better in lower extremity joint adaptability, sensory feedback, and load decentralization ability than ordinary college students. It might be attributed to the fact that ordinary college students do not have the same soccer experience or training. A recent study used a 3D-MOT task in high-intensity interval training (HIIT) to explore the effect on perceptual-cognitive performance and reported that the combination training resulted in task-specific benefits, which confirms our findings (Park et al., 2021). Romeas et al. (2016) reported that long-term MOT training improves athletic performance and decision-making ability of soccer players. Various studies have used MOT tasks for optokinetic simulation training, and the results showed that it improved posture stability for patients with motor coordination disorders such as stroke and hemiplegia (Chen et al., 2021; Komagata et al., 2021). Thus it is speculated that long-term MOT training may positively affect motor stability and improve inter-joint coordination. These findings can form a basis for designing further studies to explore application of perceptual-cognitive training in special populations with dual-task requirements. Notably, although soccer players had better coordination, the MOT task could affect their inter-joint coordination. Long-term training may improve dual-task processing ability and ensure stable coordination of soccer players but cannot eliminate the interference of MOT tasks.

The present study had some limitations. The sample size was not calculated for this study. Another limitation is that we have no idea how much these athletes train, and the ordinary students were not matched for any other characteristic. Further studies should be conducted to explore the effects of the level of training and both groups should be matched for all characteristics that may affect cognitive function.

## Conclusion

The findings showed that neuromuscular activity of individuals under perceptual-cognitive task interference cannot effectively control the challenging dual-task movement and the perceptual-cognitive task can adversely affect inter-joint coordination of soccer players and college students, mainly the hip and knee coordination. Although soccer players had better coordination, the MOT task could affect their inter-joint coordination. The findings for the study provide a theoretical basis for improving individual inter-joint coordination and reducing the risk of injury.

## Data availability statement

The original contributions presented in the study are included in the article/supplementary material, further inquiries can be directed to the corresponding author.

## Ethics statement

The studies involving human participants were reviewed and approved by the University of Soochow research ethics committee has approved the study (no. ECSU-2019000209). The patients/participants provided their written informed consent to participate in this study.

## Author contributions

YR provided conceptualization, made formal analysis, and wrote the original draft. CW modified and corrected the text expression, reviewed, and edited. AL provided decisive advice on the key steps of each experiment implementation and article revision, in charge of supervision. All authors made substantial contributions and participated in the study design, data collection, and result analysis together. We revised different versions of the paper together, and the final submitted version was approved by all authors.

## Funding

This research did not receive any specific grant from funding agencies in the public, commercial, or not-for-profit sectors.

## Conflict of interest

The authors declare that the research was conducted in the absence of any commercial or financial relationships that could be construed as a potential conflict of interest.

## Publisher’s note

All claims expressed in this article are solely those of the authors and do not necessarily represent those of their affiliated organizations, or those of the publisher, the editors and the reviewers. Any product that may be evaluated in this article, or claim that may be made by its manufacturer, is not guaranteed or endorsed by the publisher.
